# Association of plant and animal protein intake with sleep quality and quality of life in hemodialysis patients: a multicenter cross-sectional study

**DOI:** 10.3389/fnut.2024.1458560

**Published:** 2024-11-12

**Authors:** Saber Jafari Maskouni, Hossein Bavi Behbahani, Meysam Alipour, Ahmad Zare Javid, Fatemeh Fayazfar, Pardis Tofighzadeh, Shiva Shokri, Sara Keramatzadeh, Haleh Soltaniyan Dehkordi, Morteza Sharifat, Siavash Babajafari Esfandabad, Shokouh Shayanpour

**Affiliations:** ^1^Department of Nutrition, Jiroft University of Medical Sciences, Jiroft, Iran; ^2^Department of Nutrition, Shoushtar Faculty of Medical Sciences, Shoushtar, Iran; ^3^Nutrition and Metabolic Diseases Research Center, Clinical Sciences Research Institute, Ahvaz Jundishapur University of Medical Sciences, Ahvaz, Iran; ^4^Student Research Committee, School of Nutrition and Food Science, Shiraz University of Medical Science, Shiraz, Iran; ^5^Student Research Committee, Ahvaz Jundishapur University of Medical Sciences, Ahvaz, Iran; ^6^Student Research Committee, Shoushtar Faculty of Medical Sciences, Shoushtar, Iran; ^7^Nutrition Research Center, School of Nutrition and Food Science, Shiraz University of Medical Science, Shiraz, Iran; ^8^Department of Internal Medicine, Chronic Renal Failure Research Center, Ahvaz Jundishapur University of Medical Sciences, Ahvaz, Iran

**Keywords:** dietary protein, animal protein, plant protein, sleep quality, quality of life, hemodialysis

## Abstract

**Background:**

The current study aimed to evaluate the association between the intake of plant-based protein, animal-based protein, total protein, and the ratio of plant to animal protein with sleep quality and quality of life in patients undergoing hemodialysis.

**Methods:**

In this cross-sectional study, 479 adult patients undergoing dialysis for a minimum of 3 months were included. The dietary intake was calculated using information from a validated 168-item semi-quantitative food frequency questionnaire. Quality of life (QOL) was assessed using the Kidney Disease Quality of Life Short Form (KDQOL-SF 1.3). and the Pittsburgh Sleep Quality Index (PSQI) was used to assess sleep quality.

**Results:**

In this study, the mean age of the participants was 58.18 years (± 14.25 years), with the majority being male (58.2%). After adjusting for potential confounders, significant positive associations were observed between total protein intake (*β* = 0.12, *p* = 0.03) and quality of life (QOL). Conversely, there were significant negative associations between the ratio of plant to animal protein intake (*β* = −0.94, *p* < 0.01) and QOL. Furthermore, significant negative associations were found between total protein intake (*β* = −0.02, *p* < 0.05) and animal protein intake (*β* = −0.19, *p* < 0.05) with poor sleep quality. Additionally, there were significant positive associations between the ratio of plant to animal protein intake (*β* = 0.188, *p* < 0.05) and poor sleep quality.

**Conclusion:**

Increased consumption of animal protein is associated with improved sleep quality and Quality of Life (QOL) in patients undergoing hemodialysis (HD). Further research, especially prospective studies, is required to confirm these associations.

## Background

Chronic kidney disease (CKD) can progress to an irreversible stage called end-stage renal disease (ESRD), affecting millions worldwide (1, 2). Hemodialysis, often abbreviated as HD, is the main treatment for people with end-stage kidney disease (ESRD). Despite progress in treatment, many patients with end-stage kidney disease still face a high mortality risk (3).

Sleep is essential for human beings, occupying a significant portion of the lifespan and playing a critical role in the restoration and maintenance of various physiological systems such as the immune system, brain metabolism, endocrine functions, and metabolic processes (4–6). sleep problems are linked to imbalance of energy and hormonal disturbances, inflammation and inflammatory disease, such as obesity, diabetes, cardiovascular disease and decline in kidney function and increase chronic kidney disease (7–12).

quality of life (QOL) reflects individual satisfaction with life based on expectations, goals, relationships, independence, and overall well-being (13). living with HD can be difficult, as many patients experience uncomfortable symptoms like constipation, fatigue, and trouble sleeping. These challenges can make it difficult to manage daily tasks and enjoy life to the fullest and negatively impact their emotional well-being and overall quality of life (QOL) (14).

Between 41 and 85 percent of HD patients experience sleep difficulties as common consequences (15). Potential reasons of sleep disturbances include hypertension, the morning dialysis shift, blood gases, blood creatinine and urea, anemia, and stressful lifestyles (8, 16, 17). Sleep disruption affects HD patients in a variety of ways and is an emerging risk factor that can predict their quality of life (QOL) and mortality (18).

Also, Research shows that hemodialysis (HD) patients experience a lower quality of life (QOL) and experience a high rate of DALYs (Disability-adjusted life years) due to factors like reduced quality of life, comorbidities, and long-term dependency on dialysis compared to the general population (19, 20).

Studies show that diet and nutritional status play critical roles in both the health complications and quality of life of HD patients, with protein intake being one of the most important dietary factors to consider (21, 22). Inadequate protein intake is a major contributor to malnutrition, especially among elderly patients whose bodies have a harder time utilizing protein (23). Inadequate protein intake in HD patients not only increases inflammation and worsens existing complications, but also directly impacts their physical and mental well-being, creating a vicious cycle (19). For HD patients with pre-existing kidney damage or certain health conditions, high protein intake can be particularly detrimental, leading to significant increases in urea and creatinine and potentially triggering severe uremic symptoms (24). The Kidney Disease Outcomes Quality Initiative (KDOQI) recommends a daily protein intake of 1.0–1.2 grams per kilogram of body weight for individuals undergoing hemodialysis (HD) to help them maintain a stable nutritional status (25).

New research suggests the type of protein (plant or animal) HD patients eat might affect their kidney function and risk of complications (26). Depending on individual circumstances and specific medical advice, some patients may need to adjust their protein intake, including considering different plant-based and animal-based sources, to manage their serum phosphorus and potassium levels (27). To ensure they get all the essential amino acids their bodies cannot produce, some dietary guidelines recommend that hemodialysis patients source at least half of their protein from animal products like meat, poultry, fish, and eggs (28, 29). Researchs now suggests that plant-based diets and increased plant-protein intake can positively impact and may be more advantageous for HD patients compared to animal proteins. Studies have observed improved health outcomes and even associated higher fruit and vegetable consumption with lower mortality rates (30–32). A long-term study in HD patients found a plant-based diet did not raise potassium levels and seemed to improve their nutritional status (33). Furthermore, recent studies show plant-based diets can be safe and nutritious for HD patients, with diverse plant protein sources providing enough quantity and quality (34, 35). Studies suggest plant-based proteins reduce acid load and inflammation compared to animal proteins, potentially supporting kidney health and overall well-being. Red and processed meats, high in saturated fat and sodium, may worsen existing health conditions (36).

Previous research has established a connection between protein sources and the risks associated with chronic kidney disease (CKD). However, there is a notable lack of studies investigating the impact of these protein sources on sleep quality and quality of life among hemodialysis (HD) patients. This study aims to address this gap by examining the relationship between plant-based and animal-based protein intake and both sleep quality and overall quality of life in HD patients.

## Methods

### Study population and ethical considerations

We conducted a multi-center cross-sectional study on 479 HD patients in 8 Hemodialysis centers (5 Government centers and 3 Private centers) in Ahvaz, Shiraz, and Shushtar cities, Iran. Patients were included in the study if they were ≥ 18 years old, alert, and receiving HD for at least 6 months.

We excluded patients with enteral or parenteral feeding, cognitive or communication problems, severe neurological or mental disorders, active neoplastic disease, severe alcohol or drug addiction, major amputation (lower/upper extremities), diagnosis of cancer, acute or chronic pancreatitis, irritable bowel syndrome, acute or chronic pancreatitis, hepatic insufficiency, incomplete questionnaires, and their daily energy intake was less than 800 kcal/d or above 4,200 kcal/d (37).

This study was conducted according to the guidelines laid down in the Declaration of Helsinki and all procedures involving human patients were approved by the Ethics Committee of Shoushtar Faculty of Medical Sciences in Shoushtar, Iran (Registration no: IR.SHOUSHTAR.REC.1403.030). Written informed consent was obtained from all subjects.

### Assessments and measurements

#### Dietary assessment

A validated 168-item semi-quantitative FFQ, developed and validated for the Iranian population, was used to assess dietary intake (37). Participants were asked by researcher’s assistance to indicate the frequency of their consumption of each food item over the previous year, categorizing their intake as daily, weekly, or monthly. The reported frequency of each food item was adjusted to a daily intake value. The portion sizes of the foods consumed were converted into grams using standard household measurement equivalents (38). Due to the limitations of the Iranian food composition table (FCT), which only covers basic raw materials and a few nutrients, the US Department of Agriculture (USDA) FCT was used to calculate energy and nutrient intake for most foods and beverages. The Iranian FCT was only used for specific items not listed in the USDA FCT, such as “kashk” (39).

A dietary analysis was conducted using Nutritionist IV software to determine the total energy intake, plant-based protein, animal-based protein, total protein, and the ratio of plant to animal protein, macronutrient profile, and micronutrient composition.

#### Quality of life and sleep quality assessment

We used the “Kidney Disease Quality of Life” tool (KDQOL-36), a 36-item questionnaire, to measure Health-related quality of life. This questionnaire comprises 36 items divided into two principal sections: 12 generic items that evaluate overall mental and physical status, and 24 items specific to chronic kidney disease (CKD), which assess symptoms, effects, and the burden of the disease. The average scores for the five subscales range from 0 to 100, with higher scores indicating a better quality of life (QOL). The Pittsburgh Sleep Quality Index (PSQI) questionnaire was used to evaluate sleep quality (40). This tool provides a comprehensive assessment of sleep by evaluating seven key aspects: subjective sleep quality, sleep latency, sleep duration, habitual sleep efficiency, sleep disturbances, use of sleep medication, and daytime dysfunction. This questionnaire uses 19 questions to assess sleep quality. Scores range from 0 to 21, with lower scores indicating better sleep and higher scores indicating poorer sleep. A PSQI score of less than 5 indicated normal sleep, while a score exceeding 5 indicated poor sleep.

#### Anthropometric measurements

Following a dialysis session, dry weight was ascertained to the nearest 100 g utilizing digital scales, while individuals were attired in minimal clothing and devoid of footwear. This measurement protocol was adopted provided clinical evaluation did not reveal any indicative signs of hypovolemia or hypervolemia. Height was assessed without footwear, with individuals in a standing posture, achieving an accuracy of 0.1 cm. Body Mass Index (BMI) was computed by dividing the dry weight in kilograms by the square of height in meters. We employed International Physical Activity Questionnaire (IPAQ) to assess physical activity levels (41). The data were converted to metabolic equivalent hours per day (MET.h.d) for analysis.

#### Assessment of other variables

Demographic Characteristics (age, sex, marital status, employment status), dialysis history (vintage, frequency, duration), Fluid intake, urine volume, urea reduction ratio (URR), medication prescriptions and primary cause of renal failure were obtained from medical records.

#### Sample size

The G*Power 3.1.9.4 software was then used to calculate the minimum sample size required for the study. In this regard, the minimum sample size reached with statistics (i.e., significance = 0.05; power = 0.95, and effect size = 0.33) from a related previous study (42) was 389. Considering the withdrawal rate of 23%, 479 subjects were recruited.

### Data analysis and accessibility

Statistical analyses were performed using IBM SPSS Statistics version 24 (Chicago, IL, USA). *p* value ≤0.05 was considered statistically significant. To assess whether each variable followed a normal distribution, the Kolmogorov–Smirnov test was applied. Continuous variables were expressed as the mean ± standard deviation, while categorical variables were reported as percentages. Categorical variables were analyzed with the Chi-square test, while one-way ANOVA examined differences in continuous variables (total protein intake, plant protein intake, animal protein intake, and plant-to-animal protein ratio) across quartiles defined by protein intake levels. Linear regression analysis was conducted to assess the association between total, ratio, and type of dietary protein intake and sleep quality and quality of life (QOL) in crude and multivariable-adjusted models. In the first adjusted model, the confounding effects of center type, city, age, sex, diabetes, hypertension, job, marital status, education, income status, smoking, BMI, physical activity, and energy intake were controlled. Model 2 was additionally controlled for to dialysis vintage, dialysis time, frequency of hemodialysis sessions, fluid intake, urine volume, and medication prescriptions (Corticosteroids, Sevelamer hydrochloride, Calcium carbonate, Calcitriol, furosemide, lipid drugs). To provide a measure of effect size, Cohen’s f^2^ was calculated, with values of 0.1, 0.25, and 0.4 corresponding to small, medium, and large effects, respectively (43).

## Results

**Figure 1:**
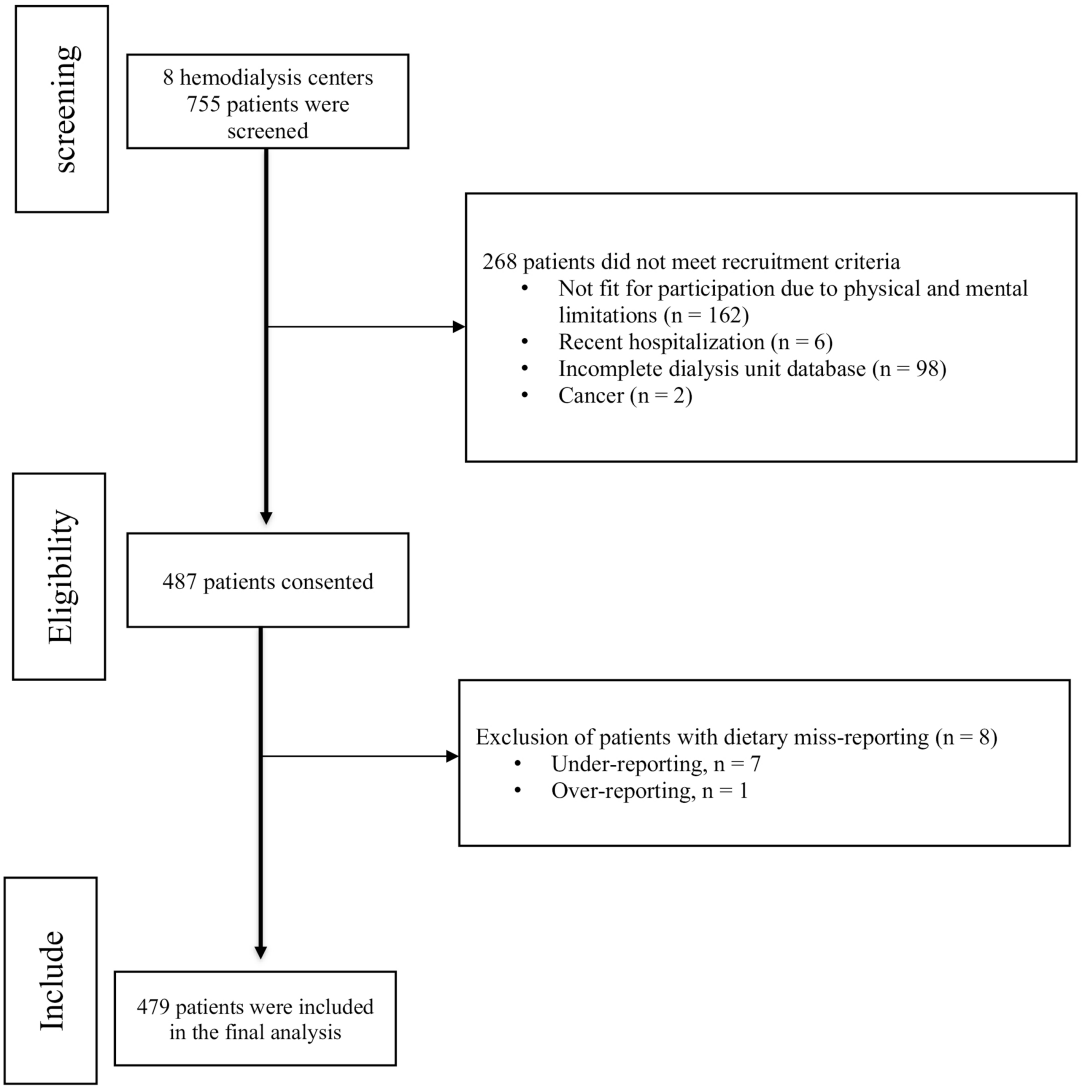
Participant flow chart.

As shown in Figure 1, out of the 755 patients evaluated across 8 hemodialysis centers, 268 were excluded from the study for various reasons. Consequently, a total of 487 patients consented to participate in the study. However, due to dietary misreporting, eight patients were excluded from the final analysis. The mean ± SD age of 479 HD patients who contributed to the current study was 58.18 ± 14.25 years. Most patients were men (58.2%), married (74.5%), either housekeeper (35.4%) or retired (23.8%). 43.7% patients’ have diabetes and most patients were high blood pressure (74.9%).

### General characteristics of the patients among quartiles of the total, plant, animal and ratio of plant to animal protein intake

The following analysis presents the general demographic and clinical characteristics of hemodialysis patients categorized into quartiles based on their total, plant, animal protein intake, and the ratio of plant to animal protein intake, as depicted in Table 1. As evident from Tables 1, a noteworthy observation is the pronounced male predominance in the highest quartiles of total, plant, and animal protein intake compared to the lower quartiles (*p* < 0.001 for all quartiles of protein intake). Additionally, there was a higher prevalence of employment and lower age among individuals in the upper quartiles of total, plant, and animal protein intake (*p* < 0.01).

**Table 1:** The baseline characteristics of study population across quartiles of total, plant, animal and the ratio of plant to animal protein intake (*n* = 479).

Characteristics, mean (SD) or N (%)	Total protein		*p*-value	Plant protein		*P*-value	Animal protein		*P*	the ratio of plant to animal protein		*P*-value
	Q1 (*n* = 119)	Q4 (*n* = 119)		Q1 (*n* = 119)	Q4 (*n* = 120)		Q1 (*n* = 119)	Q4 (*n* = 120)		Q1 (*n* = 119)	Q4 (*n* = 120)	
BMI	24.85 ± 5.63	24.28 ± 5.43	0.52	24.0 ± 5.0	24.0 ± 5.0	0.61	25 ± 4	24 ± 5	0.30	24.06 ± 5.28	24.74 ± 4.90	0.34
Age, year	58.18 ± 14.25	48.09 ± 15.04	<0.001	56 ± 14	49 ± 15	<0.001	56 ± 14	49 ± 14	<0.001	52.0 ± 14.0	53.0 ± 14.0	0.29
Frequency dialysis per week, time/week	2.91 ± 0.60	2.79 ± 0.42	0.34	2.90 ± 0.59	2.77 ± 0.47	0.20	2.85 ± 0.54	2.78 ± 0.43	0.36	2.83 ± 0.55	2.80 ± 0.45	0.28
Dialysis time, hours	3.69 ± 0.46	3.65 ± 0.54	0.82	3.66 ± 0.49	3.70. ± 0.46	0.75	3.70 ± 0.47	3.65 ± 0.56	0.82	3.61 ± 0.52	3.69 ± 0.40	0.21
Fluid intake, mL	1071.47 ± 738.27	1368.17 ± 1026.38	0.03	1,037 ± 726	1,502 ± 1,489	<0.001	1,196 ± 902	1,226 ± 714	0.39	1,062 ± 764	1,302 ± 1,379	0.27
PA, met-min/wk	177.62 ± 361.60	863.15 ± 2898.92	0.07	400 ± 2,554	653 ± 1,500	0.49	199 ± 414	682 ± 2,696	0.35	576 ± 2,695	256 ± 473	0.21
Physical activity, N (%)			0.10			0.20			0.41			0.11
Low	107 (89)	87 (73)		107 (89)	87 (73)		104 (87)	94 (78)		102 (85)	99 (82)	
Moderate	12 (10)	27 (23)		11 (9)	29 (24)		15 (12)	23 (19)		14 (11)	21 (17)	
High	0 (0)	5 (5)		0 (0)	4 (3)		0 (0)	3 (2)		3 (2)	0 (0)	
Center type (N) (%)			0.93			0.28			0.28			0.21
Governmental	78 (65)	75 (63)		82 (68)	73 (60)		76 (63)	80 (66)		82 (68)	68 (56)	
Private	41 (34)	44 (36)		37 (31.1)	47 (39)		43 (36)	40 (33)		37 (31.1)	52 (43)	
Male (N) (%)	49 (41)	93 (78)	<0.001	47 (39)	93 (77)	<0.001	58 (69)	89 (74)	<0.001	70 (58)	70 (58)	0.99
SMOKER (N) (%)	7(5)	46 (9)	0.12	7 (5)	15 (12)	0.37	7 (5)	17 (14)	0.18	12 (10)	8 (6)	0.49
Urine volume, 500 mL >	94 (78)	79 (66)	0.04	94 (78)	74 (61)	0.01	89 (74)	75 (62)	0.04	83 (69)	84 (70)	0.77
Diabetes (n, %)	59 (49)	40 (33)	0.05	82 (68)	83 (69)	0.02	53 (44)	45 (37)	0.14	57 (47)	53 (44)	0.69
Hypertension (n, %)	87 (73)	86 (72)	0.61	82 (68)	83 (69)	0.04	93 (78)	94 (78)	0.18	89 (74)	92 (976)	0.94
Job, N (%)			<0.001			<0.001			0.002			0.95
Unemployed	14 (11)	23 (19)		15 (12)	26 (21)		18 (15)	24 (20)		18 (15)	18 (15)	
Housekeeper	62 (52)	23 (19)		65 (54)	26 (21)		54 (45)	26 (21)		41 (33)	46 (38)	
Retired	27 (22)	23 (19)		24 (20)	26 (21)		27 (22)	22 (18)		27 (22)	28 (23)	
Employee	6 (5)	17 (14)		3 (2)	15 (12)		7 (5)	11 (9)		7 (5)	11 (9)	
Self-employment	7 (5)	24 (20)		9 (7)	21 (17)		9 (7)	25 (20)		18 (15)	13 (10)	
Others	3 (2)	9 (7)		3 (2)	6 (5)		4 (3)	12 (10)		8 (6)	4 (3)	
Marital status, N (%)			0.004			0.12			0.006			0.61
Married	97 (81)	85 (71)		94 (78)	83 (69)		96 (80)	89 (73)		87 (73)	94 (78)	
Single	11 (9)	26 (21)		14 (11)	23 (19)		12 (10.1)	22 (18)		20 (16)	18 (15)	
Divorced	3 (2)	7 (5)		4 (3)	4 (3)		1 (0)	9 (7)		8 (6)	3 (2)	
Dead spouse	8 (6)	1 (0)		7 (5)	7 (5)		10 (8)	1 (0)		4 (3)	5 (4)	
Education, N (%)			0.01			0.15			0.16			0.52
<12 years	100 (84)	84 (70)		99 (83)	87 (72)		102 (85)	89 (74)		92 (77)	96 (80)	
≥12 years	19 (15)	35 (29)		20 (16)	33 (27)		17 (14)	31 (25)		27 (22)	24 (20)	
Income status, N (%)			0.39			0.77			0.14			0.60
<5 million rials	45 (37)	39 (32)		41 (34)	38 (31)		41 (34)	40 (33)		43 (36)	38 (31)	
5–10 million rials	45 (37)	37 (31.1)		49 (41)	42 (35)		57 (47)	43 (35)		40 (33)	47 (39)	
10–20 million rials	24 (20)	32 (26)		25 (21)	31 (25)		17 (14)	26 (21)		28 (23)	28 (23)	
>20 million rials	5 (4)	11 (9)		4 (3)	9 (7)		4 (3)	11 (9)		8 (6)	7 (5)	
Medication prescriptions												
Calcium carbonate 500 mg, time/day	1.19 ± 1	1.06 ± 1.80	0.29	1.12 ± 1.75	1.27 ± 1.71	0.29	1.26 ± 1.82	1.02 ± 1.60	0.32	1.04 ± 1.51	1.40 ± 1.78	0.27
Sevelamer hydrochloride 800 mg, time/day	0.70 ± 1.18	0.87 ± 1.49	0.55	0.67 ± 1.19	0.84 ± 1.46	0.34	0 ± 1	0 ± 1	0.78	0.84 ± 1.32	0.91 ± 1.55	0.04
Calcitriol 0.25 mcg, time/day	0.61 ± 0.98	0.50 ± 1.00	0.14	0.72 ± 1.13	0.67 ± 1.14	0.94	0 ± 1.03	0 ± 1.00	0.46	0.57 ± 1.01	0.60 ± 1.01	0.09
Furosemide time/day	0.35 ± 0.83	0.39 ± 0.93	0.49	0.42 ± 0.89	0.23 ± 0.61	0.04	0 ± 0	0 ± 1.06	0.21	0.46 ± 0.98	0.25 ± 0.68	0.19
Corticosteroids, N (%)	7 (5)	3 (2)	0.62	7 (5)	4 (3)	0.46	4 (3)	4 (3)	0.29	7 (5)	5 (4)	0.80
Lipid-lowering drugs, N (%)	26 (21)	13 (10)	0.07	28 (23)	14 (11)	0.06	22 (18)	17 (14)	0.54	23 (19)	20 (16)	0.93
Kt/V	1.31 ± 0.56	1.15 ± 0.42	0.01	1.28 ± 0.53	1.13 ± 0.4	0.006	1.32 ± 0.61	1.14 ± 0.41	0.02	1.22 ± 0.55	1.27 ± 0.52	0.83
URR (%)	34.09 ± 31.57	40.79 ± 30.11	0.26	36.11 ± 30.62	36.55 ± 30.05	0.84	32.65 ± 31.34	41.28 ± 29.38	0.16	38.95 ± 30.3	33.61 ± 31.48	0.50

Data for quantitative variables are presented as means ± SD, obtained from ANOVA. Data for qualitative variables are presented as frequencies (percentages) and analyzed using chi-square tests. PA, physical activity; BMI, body mass index.

Furthermore, patients in the top quartiles of total and plant protein intake demonstrated a notable increase in fluid intake, while those in the lowest quartiles of total, plant, and animal protein intake exhibited a significantly higher percentage of urine volume (>500 mL) (*p* < 0.05). Notably, patients in the lowest quartiles of total and animal protein intake were more likely to be married compared to those in the top quartiles (*p* < 0.005). Moreover, higher quartiles of total protein intake were associated with a significantly higher percentage of individuals with a higher education level (*p* < 0.05) compared to the lower quartiles. Patients in the upper quartile of plant protein intake had a significantly lower furosemide consumption per day (*p* < 0.001) compared to those in the bottom quartile, whereas patients in the lowest quartile of the plant-to-animal protein ratio exhibited the lowest furosemide consumption per day (*p* < 0.001) compared to those in the bottom quartile. Additionally, concerning the etiology of End-Stage Renal Disease (ESRD), individuals in the top quartile of plant protein intake had a lower incidence of diabetes mellitus (*p* < 0.05). No notable differences were identified in other characteristics across the quartiles for all types of protein intake. Also, there was a lower Kt/V among individuals in the upper quartiles of total, plant, and animal protein intake (*p* < 0.01).

### Dietary intakes across quartiles of the total, plant, and animal proteins intake in HD patients

Table 2 presents the dietary intake across quartiles of total, plant, and animal protein intake. Notably, certain protein sources such as poultry, red meat, processed meat, fish, and low-fat dairy exhibited an increase in consumption with ascending quartiles of total protein intake, albeit only the increase in poultry consumption achieved statistical significance (*p* < 0.05). Additionally, there was a significant rise in total energy intake, carbohydrate consumption, fat intake, and fat percentage, alongside a notable decrease in carbohydrate percentage, as total protein intake quartiles increased (*p* < 0.05). Conversely, intake of vegetables and vegetable oils showed a significant decrease, while whole grain consumption exhibited a significant increase across quartiles of total protein intake.

**Table 2:** Dietary intakes across quartiles of total, plant, animal and the ratio of plant to animal protein intake (*n* = 479).

Characteristics, mean (SD) or N (%)	Total protein		*P*-value	Plant protein		*P*-value	Animal protein		*P*-value	The ratio of plant to animal protein		*P*-value
	Q1 (*n* = 119)	Q4 (*n* = 119)		Q1 (*n* = 119)	Q4 (*n* = 120)		Q1 (*n* = 119)	Q4 (*n* = 120)		Q1 (*n* = 119)	Q4 (*n* = 120)	
Food groups												
Refined grain (g/d)	198 ± 81	153 ± 86	<0.001	187 ± 79	163 ± 99	0.157	193 ± 97.05	149 ± 66	0.001	157 ± 67	178 ± 108.01	0.018
Whole grains (g/d)	32 ± 56.41	56 ± 65	<0.001	22 ± 30	74 ± 74	<0.001	59 ± 74	42 ± 51	0.09	27.02 ± 32	82 ± 78	<0.001
Beans (g/d)	63 ± 47.09	57 ± 49	0.17	62 ± 46.01	58 ± 47.02	0.283	54 ± 41	55 ± 47	0.757	56 ± 39	51 ± 39.08	0.268
Nuts (g/d)	8.06 ± 13.20	6.15 ± 7.55	0.019	8.56 ± 13.19	7.24 ± 10.48	0.696	8.33 ± 14.27	6.66 ± 8.58	0.017	8.51 ± 14.31	8.14 ± 12.60	0.908
Red meat (g/d)	5.57 ± 5.19	5.98 ± 5.57	0.557	7.32 ± 7.40	4.81 ± 4.60	0.01	4.96 ± 4.73	7.54 ± 7.15	0.005	8.28 ± 7.38	4.19 ± 3.58	<0.001
Procced meat (g/d)	1.64 ± 3.34	2.44 ± 3.63	0.361	1.99 ± 4.21	1.93 ± 3.27	0.872	1.25 ± 2.46	2.06 ± 5.07	0.076	2.47 ± 4.26	0.94 ± 1.76	0.009
Poultry (g/d)	19 ± 22	32 ± 35.03	<0.001	31 ± 34	18 ± 18	0.001	10 ± 11	42 ± 38	<0.001	48.45 ± 39.12	8.54 ± 7.26	<0.001
Fruits (g/d)	129 ± 100	102.03 ± 82	0.069	121 ± 95	104 ± 84	0.138	130 ± 120	103 ± 77	0.149	105.07 ± 69	122 ± 121	0.367
Vegetables (g/d)	56.09 ± 41	49 ± 42	0.025	56.01 ± 42	45 ± 35	0.051	54 ± 39	52 ± 43	0.945	55 ± 42	47 ± 39	0.410
Vegetable oils (g/d)	6.31 ± 5.00	3.45 ± 3.03	0.001	6.43 ± 5.06	3.35 ± 2.60	<0.001	4.99 ± 4.20	3.96 ± 3.28	0.062	5.42 ± 4.61	3.65 ± 2.85	0.003
Soft drink (g/d)	11 ± 23	17 ± 30	0.06	13 ± 26	17 ± 29	0.235	10 ± 21	17 ± 30	0.048	14 ± 26	10.62 ± 23.90	0.471
Sweets and dessert (g/d)	9 ± 11	11.02 ± 13	0.370	9.39 ± 11.39	9.49 ± 11.65	0.677	8.81 ± 10.49	11.75 ± 14.95	0.097	9.92 ± 12.26	7.78 ± 9.69	0.111
Potato (g/d)	5.13 ± 5.96	5.09 ± 7.37	0.077	6.22 ± 9.56	4.93 ± 6.80	0.5	5 ± 5	5 ± 7	0.064	5.91 ± 7.93	5.38 ± 6.46	0.09
Low fat dairy (g/d)	10 ± 26	20 ± 50	0.160	13 ± 27	15 ± 24	0.461	8.83 ± 18.71	25 ± 65	0.007	24 ± 56	10.02 ± 15	0.026
High fat dairy (g/d)	39 ± 41	34 ± 32	0.347	36 ± 40	31 ± 27	0.046	29 ± 33	36 ± 34	0.257	39 ± 39	23 ± 26.09	<0.001
Fish (g/d)	5.66 ± 7.41	9.19 ± 13.28	0.064	8.02 ± 12.05	6.09 ± 7.46	0.455	4.22 ± 5.90	11.54 ± 15.14	<0.001	12.15 ± 15.02	4.26 ± 5.46	<0.001
Egg (g/d)	12.78 ± 13.28	9.79 ± 10.20	0.112	13.38 ± 13.86	8.06 ± 7.73	<0.001	10.67 ± 11.40	10.58 ± 10.98	0.049	12.92 ± 14.13	7.78 ± 8.88	0.001
Nutrients												
Total energy (kcal/d)	1,335 ± 337	3,234 ± 712	<0.001	1,350 ± 398	3,229 ± 669	<0.001	1,665 ± 602.08	2,863 ± 878	<0.001	2,114 ± 1,005	2,182 ± 848	0.021
Carbohydrate (g/d)	203 ± 61	472 ± 136	<0.001	189 ± 52.06	502 ± 109	<0.001	276 ± 119	395 ± 140	<0.001	275.43 ± 134.06	394.69 ± 155.96	<0.001
Fat (g/d)	41 ± 16	97 ± 42	<0.001	46.07 ± 23	88 ± 45	<0.001	41 ± 19	93 ± 41.05	<0.001	74.83 ± 42.24	54.21 ± 30.98	<0.001
Carbohydrates percent	60 ± 8	58 ± 9	0.021	56 ± 10	62 ± 9	<0.001	64 ± 10	54 ± 8	<0.001	52 ± 7.12	66 0.78 ± 9.89	<0.001
Fat percent	27 ± 8	58 ± 9	<0.001	29 ± 8	24.05 ± 9	<0.001	23.44 ± 9.24	29.05 ± 7.45	<0.001	31 ± 7.11	20.87 ± 8.55	<0.001

The data are presented as “mean ± SD.” The significant difference based on One-way ANOVA (*p* < 0.05).

Furthermore, individuals within the highest quartile of plant protein intake demonstrated significantly elevated consumption of whole grains and reduced intake of red meat, poultry, vegetable oils, high-fat dairy, and eggs compared to lower quartiles (*p* < 0.05). Concurrently, there was a significant increase in total energy intake, carbohydrate consumption, fat intake, and carbohydrate percentage, with a corresponding decrease in fat percentage across quartiles of plant protein intake (*p* < 0.05).

Among different quartiles of animal protein intake, individuals within the top quartiles exhibited significantly higher consumption of soft drinks, red meats, poultry, fish, low-fat dairy, energy, fat, carbohydrates, and percentage of fat (*p* < 0.05), while demonstrating significantly lower intake of refined grains, nuts, eggs, and carbohydrate percentage compared to lower quartiles (*p* < 0.001).

In addition, across varying quartiles of the plant-to-animal protein ratio, individuals within the highest quartiles displayed significantly reduced consumption of red meats, processed meats, poultry, fish, eggs, low-fat dairy, high-fat dairy, vegetable oils, fat, and percentage of fat (*p* < 0.05), while exhibiting significantly increased intake of refined grains, whole grains, total energy intake, carbohydrates, and carbohydrate percentage compared to lower quartiles (*p* < 0.001).

### Association of QOL and sleep quality with different types of dietary protein intake in HD patients

The outcomes of linear regression analysis examining the relationship between Quality of Life (QOL), sleep quality, and various types of dietary protein intake among Hemodialysis (HD) patients are delineated in Table 3.

**Table 3:** The association between different types of dietary protein intake and sleep quality and quality of life in hemodialysis patients (*n* = 479).

	Model 0^a^			Model 1^b^			Model 2^c^		
	β (95% CI)	*P*-value	Effect size	β (95% CI)	*P*-value	Effect size	β (95% CI)	*P*-value	Effect size
Total protein									
Quality of life	0.164 (0.133, 0.195)	<0.001	0.47	0.214 (0.151, 0.277)	<0.001	0.55	0.206 (0.140, 0.272)	<0.001	0.51
Sleep quality	−0.02 (−0.03,-0.01)	<0.001	0.19	−0.024 (−0.045, −0.004)	0.021	0.30	−0.022 (−0.043, −0.001)	0.037	0.31
Animal protein									
Quality of life	0.220 (0.173, 0.266)	<0.001	0.17	0.175 (0.120, 0.229)	<0.001	0.54	0.171 (0.115, 0.226)	0.305	0.51
Sleep quality	−0.03 (−0.04,-0.01)	<0.001	0.42	−0.019 (−0.037, −0.001)	0.034	0.30	−0.19 (−0.036, −0.001)	0.039	0.31
Plant protein									
Quality of life	0.181 (0.124, 0.239)	<0.001	0.47	−0.075 (−0.189, 0.039)	0.196	0.44	−0.099 (−0.215, 0.017)	0.095	0.41
Sleep quality	−0.03 (−0.05,-0.01)	0.002	0.14	0.005 (−0.030, 0.040)	0.769	0.28	0.011 (−0.025, 0.047)	0.539	0.30
Plant/Animal protein									
Quality of life	−0.826 (−1.466, −0.185)	0.012	0.46	−0.906 (−1.509, −0.303)	0.003	0.46	−0.941 (−1.546, −0.335)	0.002	0.43
Sleep quality	0.177 (−0.14, 0.37)	0.07	0.08	0.179 (−0.011, 0.368)	0.064	0.30	0.188 (0.001, 0.375)	0.048	0.31

*p < 0.05 were considered as statistically significant. Effect size = Cohen’s f2 for linear models.*

a Model 0, linear regression analysis without adjustment.

b Model I, linear regression analysis with adjustment for center type, city, age, sex, diabetes, hypertension, job, marital status, education, income status, smoking, BMI, physical activity, and energy intake.

c Model II, linear regression analysis with correction for center type, city, age, sex, diabetes, hypertension, job, marital status, education, income status, smoking, BMI, physical activity, energy intake, dialysis vintage, dialysis time, frequency of hemodialysis sessions, fluid intake, urine volume, and medication prescriptions (Corticosteroids, Sevelamer hydrochloride, Calcium carbonate, Calcitriol, furosemide, lipid drugs).

Statistically significant positive correlations were evident between total protein intake (*β* = 0.16; *p* < 0.001), plant protein intake (*β* = 0.18; *p* < 0.001), and animal protein intake (*β* = 0.22; *p* < 0.001) with QOL. Conversely, a significant negative correlation was observed between the ratio of plant to animal protein intake (*β* = −0.82; *p* < 0.012) and QOL in model 0 (unadjusted). Upon adjustment for confounding variables including center type, city, age, sex, diabetes, hypertension, job, marital status, education, income status, smoking, BMI, physical activity, and energy intake in Model 1, statistically significant positive associations persisted between total (*β* = 0.21; *p* < 0.001) and animal (*β* = 0.17; *p* < 0.001) protein intake and QOL. Conversely, a significant negative correlation was maintained between the ratio of plant to animal protein intake (*β* = −0.90; *p* < 0.01) and QOL. However, in Model 1, the association between plant protein intake and QOL was non-significant (*β* = −0.07; *p* = 0.19). Furthermore, in Model 2, which accounted for additional confounding variables such as dialysis vintage, dialysis time, frequency of hemodialysis sessions, fluid intake, urine volume, and medication prescriptions, significant positive associations were only observed between total protein intake (*β* = 0.12; *p* = 0.03) and negative associations of the ratio of plant to animal protein intake (β = −0.94; *p* < 0.01) with QOL. However, in Model 3, the associations between animal (*β* = 0.17; *p* = 0.30) and plant protein (*β* = −0.09; *p* = 0.09) intake with QOL were non-significant.

Additionally, significant positive associations were detected between total protein intake (*β* = −0.02; *p* < 0.001), plant protein intake (*β* = −0.03; *p* < 0.01), and animal protein intake (*β* = −0.03; *p* < 0.001) with the poor sleep quality. However, the association between the ratio of plant protein to animal protein intake and the sleep quality was not statistically significant in model 0 (unadjusted) (*β* = 0.01; *p* = 0.07).

In Model 1, following adjustments for various confounding factors, including center type, city, age, sex, diabetes, hypertension, job, marital status, education, income status, smoking, BMI, physical activity, and energy intake significant negative associations were observed between total (*β* = −0.02; *p* < 0.05) and animal (*β* = −0.02; *p* < 0.05) protein intake and the poor sleep quality. Conversely, a significant negative association was found between the ratio of plant to animal protein intake (*β* = −0.90; *p* < 0.01) and the poor sleep quality. However, in Model 2, the associations between plant protein intake (*β* = 0.005; *p* = 0.769) and the ratio of plant protein to animal protein intake (*β* = 0.18; *p* = 0.064) with the score of sleep quality were not statistically significant.

Moreover, in Model 2, after adjusting for Model 1 confounding variables along with additional factors such as dialysis vintage, dialysis time, frequency of hemodialysis sessions, fluid intake, urine volume, and medication prescriptions significant negative associations were observed between total (*β* = −0.02; *p* < 0.05) and animal (*β* = −0.19; *p* < 0.05) protein intake and positive associations of the ratio of plant to animal (*β* = 0.188; *p* < 0.05) with poor sleep quality. However, in Model 3, the association between plant protein intake (*β* = 0.011; *p* = 0.539) and sleep quality was not statistically significant.

## Discussion

For patients undergoing hemodialysis (HD), a well-balanced diet, particularly adequate protein intake, significantly impacts their sleep quality and quality of life (QOL). Maintaining good nutritional status, especially ensuring sufficient dietary protein, is crucial for hemodialysis patients to experience a well sleep and quality of life (QOL). While the link between protein intake and sleep quality has been investigated in other disease and conditions, no studies have specifically investigated this association in patients undergoing hemodialysis (44–47). However, there is a few research that exploring the link between dietary protein intake and quality of life in hemodialysis patients (48–51).

Besides the amount of protein consumed, the type of protein may also influence complications and quality of life in patients undergoing HD (52, 53). This study investigates the link between protein source, sleep quality, and quality of life (QOL) in HD patients. It is the first to examine the protein-sleep quality connection and the second to explore the protein-QOL association in this population. We observed that higher total protein consumption was associated with better QOL in HD patients, while there was negative significant association between higher ratio of plant to animal protein consumption with QOL. Also, we observed that higher total and animal protein consumption was associated with better sleep quality in HD patients, while there was negative significant association between higher ratio of plant to animal protein consumption with good sleep quality.

Limited research has been conducted on the relationship between overall dietary protein consumption and quality of life (QOL) among individuals undergoing hemodialysis (HD) (23, 50, 54). Darzi et al. observed a notable positive connection between total protein intake, as well as protein derived from plant and animal sources, and QOL in HD patients (53). Similarly, Sharin et al. found a significant positive link between protein intake and the physical component of Quality of Life (QOL) in hemodialysis (HD) patients. However, they did not observe a significant association between overall nutritional status and QOL (23). Furthermore, two separate cross-sectional studies demonstrated an association between low dietary protein intake, as indicated by low serum albumin levels, and a lower quality of life in patients undergoing hemodialysis (50, 55). Individuals undergoing hemodialysis experience catabolic mechanisms related to the procedure, making it crucial to consider a higher protein intake in their diet to counteract muscle loss and reduce susceptibility to infections (56). A lack of protein intake can also lead to anemia, weakness, and fatigue, directly affecting both physical and mental quality of life (21, 57).

In contrast, based on a study by Yusop et al., individuals receiving hemodialysis (HD) treatment may experience an improved quality of life (QOL) when adhering to lower protein consumption. Notably, the study found that patients failing to achieve the recommended protein intake levels could still exhibit favorable quality of life assessments and maintain a healthier body mass index (BMI) (58). Our differing methodologies for assessing dietary intake may account for the variation in results compared to Yusop et al. While they employed a 24-h diet recall, our study utilized a Food Frequency Questionnaire (FFQ), which captures dietary patterns over a longer period and potentially provides a more comprehensive picture of habitual intake (34, 35).

Our research demonstrated a positive association between increased consumption of total and animal protein and enhanced quality of life (QOL). Our hypothesis for this outcome is that the significant price discrepancy between animal protein sources and plant protein sources in Iran may play a role (59).

In Iran, animal proteins may be less accessible due to cost, leading to a higher intake of plant proteins that are less suited to the specific needs of dialysis patients. In low-income populations, limited access to high-quality protein sources can further reduce dietary protein adequacy, contributing to malnutrition and a poorer QoL (60). One study found that higher income groups are more likely to afford and consume animal proteins such as red meat, fish, and poultry, which are typically more expensive than plant-based proteins (61).

Another possibility is that protein helps to preserve muscle mass and function. Protein is essential for muscle growth and repair, and a high protein intake can help to offset the muscle loss that can occur in HD patients. Additionally, protein can help to improve energy levels and reduce fatigue by providing the body with a source of amino acids, which can be used for energy production (62). it should be noted that animal proteins are recognized for their high biologic value, and previous studies have recommended that dialysis patients should consume a minimum of 50% high biologic value proteins whereas plant-based proteins are typically deficient in one or more essential amino acids, particularly lysine, methionine, and leucine, which are crucial for muscle protein synthesis (28, 31).

Another possibility is that plant proteins have a lower digestibility compared to animal proteins. Factors like plant cell walls, fiber, and anti-nutritional compounds (e.g., phytates and tannins) hinder protein absorption (63). This is significant in hemodialysis patients, who may already experience digestive challenges due to gastrointestinal complications related to their treatment (64). In contrast, animal proteins are generally more easily digested and absorbed, providing more immediate nutritional support. For HD patients, who already experience decreased appetite and gastrointestinal challenges, ensuring that the consumed protein is efficiently utilized is crucial for preventing malnutrition and maintaining QoL (65).

Sleep is essential for overall human health and function. Sleep deprivation is associated with a wide range of negative consequences, including cognitive impairment, immune system dysfunction, and an increased risk of chronic diseases (66, 67). Several factors influence sleep quality, including behavioral, environmental, and physiological factors (68). Diet is one often-overlooked factor that can significantly impact sleep (44). Some studies have shown that higher animal protein consumption may improve sleep quality (69). For instance, one study on older adults found that those who consumed more animal protein had deeper sleep compared to those who consumed less animal protein (44, 70). Another study that results from 2 randomized controlled trials demonstrated that consuming higher protein improved indexes of sleep in energy-restricted overweight and obese adults (71). Several mechanisms may explain how animal protein could improve sleep quality. One possibility is that animal protein is a good source of tryptophan, an amino acid that converts into the precursor of serotonin, a neurotransmitter that plays a crucial role in regulating sleep (72, 73). Additionally, animal protein provides essential amino acids required for the production of melatonin, a hormone that helps regulate the sleep–wake cycle (73, 74).

Notwithstanding the positive effects of animal-based protein consumption, it is advisable for individuals with HD to exercise caution when including such proteins in their diet (35). A concern about animal-based proteins is their potential to contribute to the accumulation of uremic toxin production, particularly indoxyl sulfate and p-cresyl sulfate, which are by-products of the metabolism of aromatic amino acids (such as tryptophan and tyrosine) (75). Chronic inflammation is a hallmark of CKD, and uremic toxins play a central role in its pathogenesis (76, 77). These toxins activate pro-inflammatory pathways, such as NF-kB and Toll-like receptors (TLRs), which in turn increase the production of inflammatory cytokines like IL-6 and TNF-*α* (78).

Considering the beneficial effects of animal-derived proteins in managing various complications in hemodialysis (HD) patients, and the impact of these complications on sleep quality and overall Quality of Life (QOL), it is reasonable to hypothesize a potential positive correlation between animal protein intake and the overall QOL in this patient population.

This study is the first to investigate the impact of different types of dietary protein on sleep quality and QOL among HD patients, effect size for quality of life was large and adjustment for potential confounders were done. For this reasons, Our results can be used to inform the development of strategies and dietary guidelines for hemodialysis patients.

However, several limitations must be acknowledged. Due to the cross-sectional nature of this study, it is not possible to determine any cause-and-effect relationships between the variables. Another point worth considering is that even though the study employed a validated Food Frequency Questionnaire (FFQ), there may still be potential inaccuracies in measuring dietary intake, along with possible recall biases. BMI has significant shortcomings, particularly when compared to more advanced methods like bioelectrical impedance analysis (BIA), which can assess body composition, including muscle. Lastly, it is important to acknowledge that multiple variables could have impacted sleep quality and Quality of Life (QOL) in this study, which the researcher was unable to control.

Although our study found a positive link between higher animal protein intake and improved sleep quality and Quality of Life (QoL), it is important to consider the potential risks associated with excessive protein consumption. Further longitudinal studies are necessary to confirm these findings and thoroughly evaluate the long-term risks linked to high consumption of animal protein. Also, future investigations should utilize randomized controlled trials (RCTs) and advanced body composition measurement methods like bioelectrical impedance analysis (BIA) to determine optimal protein intake and understand its effects in hemodialysis patients.

## Conclusion

In conclusion, the results of our study suggest that a higher intake of animal protein, in contrast to plant protein, is associated with better sleep quality and Quality of Life (QOL) among patients undergoing hemodialysis (HD).

## Data Availability

The original contributions presented in the study are included in the article/supplementary material, further inquiries can be directed to the corresponding author.
